# Modelling Niche Differentiation of Co-Existing, Elusive and Morphologically Similar Species: A Case Study of Four Macaque Species in Nakai-Nam Theun National Protected Area, Laos

**DOI:** 10.3390/ani3010045

**Published:** 2013-01-30

**Authors:** Camille N. Z. Coudrat, K. Anne-Isola Nekaris

**Affiliations:** Anthropology Centre for Conservation, Environment and Development, Department of Anthropology and Geography, Oxford Brookes University, Oxford OX3 0BP, UK; E-Mail: anekaris@brookes.ac.uk

**Keywords:** *arctoides*, *assamensis*, camera-trapping, ecological niche modelling, habitat suitability, Lao PDR, *leonina*, MaxEnt, *mulatta*, predictive modelling

## Abstract

**Simple Summary:**

We investigated the niche separation of four macaque species (*Macaca arctoides*, *M. assamensis*, *M. leonina*, *M. mulatta*) occurring within Nakai-Nam Theun National Protected Area, central-eastern Laos using the environmental niche modelling software MaxEnt. The respective suitable habitat predicted for each species reveals niche segregation between the four species with a gradual geographical distribution following an environmental gradient of, notably, temperature, precipitation, elevation and slope within the study area. This means that the four species seem adapted to different ecological conditions within the area. This information has implications for future research on these species and for their management and conservation.

**Abstract:**

Species misidentification often occurs when dealing with co-existing and morphologically similar species such as macaques, making the study of their ecology challenging. To overcome this issue, we use reliable occurrence data from camera-trap images and transect survey data to model their respective ecological niche and potential distribution locally in Nakai-Nam Theun National Protected Area (NNT NPA), central-Eastern Laos. We investigate niche differentiation of morphologically similar species using four sympatric macaque species in NNT NPA, as our model species: rhesus *Macaca mulatta* (Taxonomic Serial Number, TSN 180099), Northern pig-tailed *M. leonina* (TSN not listed); Assamese *M. assamensis* (TSN 573018) and stump-tailed *M. arctoides* (TSN 573017). We examine the implications for their conservation. We obtained occurrence data of macaque species from systematic 2006–2011 camera-trapping surveys and 2011–2012 transect surveys and model their niche and potential distribution with MaxEnt software using 25 environmental and topographic variables. The respective suitable habitat predicted for each species reveals niche segregation between the four species with a gradual geographical distribution following an environmental gradient within the study area. Camera-trapping positioned at many locations can increase elusive-species records with a relatively reduced and more systematic sampling effort and provide reliable species occurrence data. These can be used for environmental niche modelling to study niche segregation of morphologically similar species in areas where their distribution remains uncertain. Examining unresolved species’ niches and potential distributions can have crucial implications for future research and species’ management and conservation even in the most remote regions and for the least-known species.

## 1. Introduction

Environmental niche models are increasingly used to understand species’ habitat use, to study their evolutionary biogeography, and to predict their geographic distribution at regional or national scales in relation to their environment e.g., [1,2,3,4,5,6]. These models constitute useful tools to predict suitable habitat of species in areas where their distribution is little known. 

Species misidentification is a common issue during field surveys, especially so when dealing with morphologically similar species that flee when sighted e.g., [7,8,9]. Camera-traps, in contrast, can capture clear images that allow detailed examination of morphological difference between such species and result in reliable species occurrence data (necessary for environmental niche modelling). In addition, camera-traps increase the probability of records of elusive species, compared to field surveys e.g., [10,11] making the technique particularly valuable when direct data collection in the field is logistically challenging, financially restricted and time consuming. Such data are furthermore rarely readily available to the scientific community. Although the importance of camera-trap data is being acknowledged for understanding species’ ecology [12,13,14], these data have rarely been used in combination with species niche modelling e.g., [15]. Taking this further, combining camera-trapping data and species niche modelling provides a powerful technique to explore the ecological relationships of co-existing, cryptic and morphologically similar species (e.g., macaques, small cats, civets, ungulates).

In this study, we focus on macaque species. Four macaque species occur in our study area, Nakai-Nam Theun National Protected Area (NNT NPA), Laos: rhesus (*Macaca mulatta*), Northern pig-tailed (*M. leonina*), Assamese (*M. assamensis*) and stump-tailed (*M. arctoides*) macaques. Each has been previously confirmed from field sightings [16] and camera-traps (WMPA unpubl. data). Field identification confusion however can occur between species, and this is further emphasized by the unreliable and misidentification of macaques by villagers living alongside the species’ habitat [9]. Fooden [17] predicted ecological segregation across their range amongst these species, but until now this has rarely been studied in the field, and never in Laos, where all four taxa are under threat from hunting. The predictive distribution of macaque species in NNT NPA will help us understand the difference in ecological niche between these four species. We use confirmed identified camera-trap photos from 2006–2011 and field records from 2011–2012 to model their distribution using the maximum entropy (MaxEnt) statistical method.

## 2. Methods

### 2.1. Study Area

**Figure 1:**
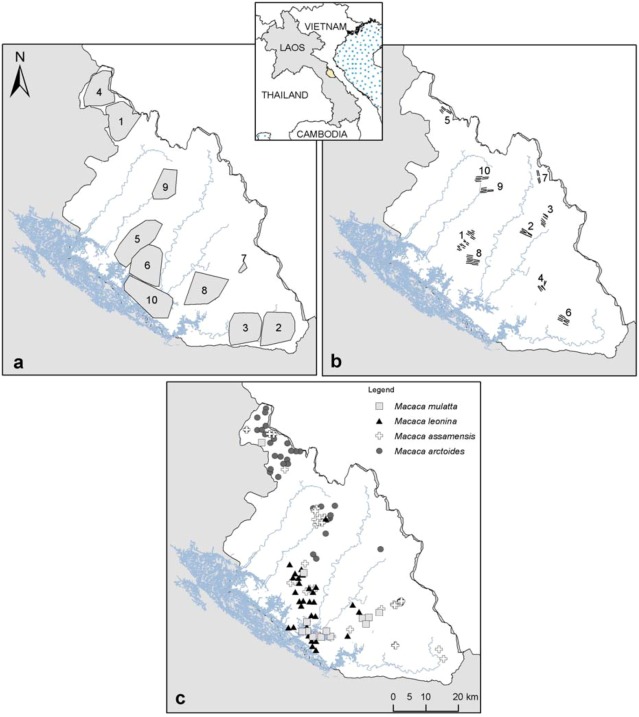
(**a**) Camera-trap sampling effort from 2006–2011 (sampling areas obtained with a minimum convex polygon around camera-traps set; *cf.* Table 1); (**b**) transect surveys from 2011–2012, total km walked, including replications are presented in Table 1; (**c**) Macaques species occurrence localities used for the models.

Nakai-Nam Theun National Protected Area (NNT NPA), ~3,500 km^2^, is located in the central-east of Laos within the Annamite mountain range (Figure 1). The area remains largely forested [18] with mixed semi-evergreen/coniferous, upper montane, dry-evergreen and wet evergreen forests [16]. Elevation throughout the NPA ranges from ~200 m to *ca*. 2,300 m a.s.l. Annual precipitation ranges from 1,865 mm to 2,620 mm and annual mean temperatures from 14 °C to 24 °C, with extremes of 4 °C to 32 °C [19].

### 2.2. Biological and Environmental Data

Occurrence data for macaque species come from camera-trap and transect survey data. From 2006 to 2011 systematic camera-trapping was carried out in the area by the Nam Theun 2-Watershed Management and Protection Authority [20,21]. Camera-trap sampling was designed as to be as representative as possible of the different habitats in NNT NPA for long-term monitoring of its wildlife [20]. We examined all photos taken over the study period and selected all photos of macaque species to identify them at species level. We identified species in light of their respective morphology, clearly observed on photos, and validated our identifications with expert opinion. In addition, CNZC conducted transect surveys in 2011–2012 within the area during which she recorded all diurnal primate species [22]. Given the difficulty to identify macaque species in the field due to poor visibility and fleeting behaviour of the animals, we only use confirmed species records (*i.e.*, involving observation of species-typical characteristics) for the analysis. Ten different sites were sampled with camera-traps from 2006 to 2011, with a total of 20,216 camera-trapping-days and ten sites were visited for transect surveys in 2011–2012, totalling 310 km walked (Table 1; Figure 1). We obtained a total of 48, 38, 34 and 14 locality points for *M. arctoides*, *M. assamensis*, *M. leonina* and *M. mulatta*, respectively (Table 2). 

**Table 1:** Details of camera-trapping and transect survey effort in Nakai Nam Theun NPA from 2006 to 2012.

Camera-trap surveys				Transect surveys			
Site # on Figure 1	Survey period	Total cameras ^a^	Camera-trap-day	Site # on Figure 1	Survey period	Nb. Transects (rep.)	km walked
1	Mar–May 06	49	2,181	1	29 Jan–2 Feb 11	20 (×1)	21
2	Oct–Nov 06	49	1,638	2	17 Feb–6 Mar 11	6 (×3) 1 (×1)	36
3	Dec 06–Feb 07	49	1,705	3	13 Mar–31 Mar 11	7 (×3) 1 (×2)	40
4	Mar–May 07	48	2,134	4	18 Jul–3 Aug 11	6 (×1)	11
5	Nov 07–Jan 08	50	2,308	5	16 Sept–28 Sept 11	6 (×1)	10
6	Jan–Mar 08	47	1,846	6	19 Oct–4 Nov 11	8 (×3)	42
7	Apr–Aug 08	32	1,687	7	11 Jan–23 Jan 12	4 (×3)	22
8	Nov 08–Jan 09	22	1,174	8	10 Feb–27 Feb 12	10 (×3)	58
9	Mar–May 09	3	183	9	12 Mar–24 Mar 12	6 (×3)	34
10	Nov–Dec 09	40	1,636	10	25 Mar–5 Apr 12	6 (×3)	35
9	Mar–May 10	45	2,405				
1	Dec 10–Jan 11	33	1,319				
**TOTAL**	**Mar 06–Jan 11**	**467**	**20,216**	**TOTAL**	**Jan 11–Apr 12**	**176**	**310**

^a^ Only one camera was set at each locality (*i.e.*, no paired cameras).

**Table 2:** Occurrence from camera-trap and transect survey data used for the distribution modelling of each species.

Species	Occurrence from camera-trap	Occurrence from confirmed sighting	Total
*M. mulatta*	14	0	**14**
*M. leonina*	31	3	**34**
*M. assamensis*	22	16	**38**
*M. arctoides*	45	3	**48**

Of a total of 114 independent images of macaque species, only two remained unidentified, while of the 55 sightings of macaques during our transect surveys, 33 remained unidentified. Species were identified by examining respective specific morphological characteristics.

We used 25 environmental variables in our models, of which 19 are derived from monthly min/max temperature and precipitation data averaged as annual trends for the period 1950–2000 (30 arc-second/1 km^2^ resolution; (http://www.worldclim.org/current/) [19]). We retrieved elevation layer from the CGIAR Consortium for Spatial Information (http://srtm.csi.cgiar.org/). Land cover data were generated by the Lao National Geographic Department in 2002 and includes 24 categories from which 14 (upper evergreen, upper mixed deciduous, pine, mixed broadleaf-coniferous, unstocked, bamboo, ray, savannah, scrub, rice paddy, rock, grassland, swamp, water bodies) are found within the boundary of NNT NPA. We obtained vegetation continuous field from the Global Land Cover Facility (http://glcf.umiacs.umd.edu/data/vcf/). We derived slope from elevation and distance from water and villages from villages and watercourse features using Euclidean distances in ArcGIS 9.3. The same geographic extent, cell size (90 m; this resolution is considered adequate given in general a >1 km^2^ home range size of macaque species; [23]) and projected coordinate system (WGS 1984 UTM Zone 48N) were selected for each layer and species localities.

### 2.3. Species Distribution Modelling

To model the potential distribution and niche of macaque species in NNT NPA, we used the maximum entropy general purpose machine learning method, which has been adapted and developed as a software (MaxEnt) for species distribution modelling [24,25] and identified as one of the best methods for species niche modelling [26]. MaxEnt version 3.3.3k is used to perform the analysis (http://www.cs.princeton.edu/~schapire/MaxEnt/). The method combines biological data of species occurrence (presence-only data) with environmental characteristics (e.g., GIS layers with a grid of values for the geographic area considered) to estimate the probability distribution of maximum entropy (*i.e.*, closest to uniform), subject to the set of constraints provided (*i.e.*, environmental characteristics where the species occurs) [25].

### 2.4. Model Building

For each species, we used a ten-fold cross-validation replication run type [27] where a sample of the occurrence localities is randomly allocated for test data (for model evaluation) at each of the ten replications. We set all other settings as default as they perform well and are robust for a large range of datasets tested, which our data can compare to [28].

### 2.5. Model Evaluation

We assessed the performance of our models using four methods: (i) the area under curve (AUC) of the receiver-operating characteristic (ROC) (ii) the use of a null-model [29], (iii) the Boyce Index method [30], (iv) and the jackknife validation method [31] for our smallest sample only. In addition, results outputs are interpreted in term of known ecology of the study species at National scale, published by experts (*cf*. Discussion section).

The AUC of ROC is provided in the model outputs. It is obtained by plotting *sensitivity* on the x-axis and 1-*specifici*ty on the y-axis, with *sensitivity* representing the proportion of correct prediction of actual presence (true-positive, or absence of omission), whereas 1-*specificity* is the proportion of falsely predicted presence (false-positive, or commission error) for all possible thresholds of the probability (threshold independent evaluation). In presence-only models, the AUC value represents the probability that the model scores a presence site (test locality) higher than a random background site [25]. The value ranges from 0.5 to 1−*a*/2, where *a* is the fraction of pixels covered by the species’ distribution that remains unknown, thus renders inadequate the interpretation of AUC [25,29,32]. Nevertheless, it remains the most commonly used evaluation parameter and is presented here. An AUC value closer to 1 indicates that the model predicts better than random, while a value of 0.5 indicates that the prediction is no better than random [25]. 

Given the recent critics of using AUC for presence-only model evaluation [29,32], we used other methods to evaluate the performance of the model outputs. A recently developed alternative is the null-model, which was introduced by Raes and Steege [29]. The method tests the AUC value of the model against a null distribution of expected AUC values based on random occurrence data from the geographic area considered. More concretely, it tests if the relations between species occurrence and environmental variables at these locations are stronger than expected by chance [29]. The exact same number of occurrence points available for each species (48, 38, 34 and 14; *cf.* Table 2) were randomly drawn across NNT NPA and replicated 999 times. These null-distributions were run separately in MaxEnt with the same settings detailed above. This resulted in 999 AUC values of the null-model for each of the four species. AUCs of the null-models were ranked and the position of the real species’ AUC was tested against the 95% confidence interval (CI) of the null-model’s AUC values. The species’ model is considered performing better than expected by chance if its AUC value is ≥95% CI null-model’s AUCs [29].

In addition, we apply the Boyce Index validation method [30,33] to our models. The continuous habitat suitability scores obtained from the outputs (0 to 1) are reclassified into a number *i* of bins (classes). For each class, two frequencies of pixels are calculated: the Predicted Frequency and the Expected Frequency. The Predicted Frequency is the number of occurrence points predicted by the model falling into in the class *i* divided by the total number of occurrence points. The Expected Frequency is the number of grid cells included in class *i*, divided by the total number of grid cells in the whole geographic area considered. A Predicted-to-Expected ratio is calculated for each class and a Spearman rank correlation coefficient *rho* (1-tailed test) evaluates if the ratio significantly increases as suitability increases (*p* < 0.05) [33]. Our models’ outputs were reclassified into 100 continuous classes of equal interval and we calculated a P/E ratio for each class. Statistical tests were performed with SPSS.v.17.

Finally, for the smallest sample (*M. mulatta*; *n* = 14), we followed the jackknife validation method for samples *n* < 25 described by Pearson *et al.* [31], in which it is assessed if the model successfully predicts the n left-out localities (one locality at each of the 14 replications) within the area of suitability (under the minimum training presence threshold chosen). This is assessed with a pValue based on the test statistic *D*; *D =* ΣX_i_ (1 − P_i_), where X*i* is the success-failure variable indicating if the *i*th left-out locality is included or not in the predicted area and P*i* is the probability of success [31]. The pValue is computed with *pValue compute* program [31].

### 2.6. Output Analysis

To produce a binary map (*i.e.*, suitable *vs.* non-suitable habitat), we used the “*minimum training presence logistic*” threshold, which has been commonly used when occurrence data are highly reliable, such as here, given the confirmed species identification and records in their primary habitat. This threshold has the advantage to maintain zero omission error and include all areas that are at least as suitable as those where the species is known to occur [31,34]. The final potential distribution for each species was projected (WGS 1984 UTM Zone 48N) in ArcGIS 9.3. 

We calculated mean values of environmental variables within the predicted distribution of the four species. Predicted suitable habitat of the four species is also tested pair-wise for their similarity using two different statistical tests that compare the logistic habitat suitability scores provided in MaxEnt’s model outputs: Schoener’s D [35] and *I* statistic [36]. Their value ranges from 0 (no overlap in habitat suitability) to 1 (complete overlap in habitat suitability); they are calculated using the ENMTool [36]. Range overlap is also quantified with ENMTool with the formulae (N_x,y_/min[N_x_, N_y_]), where N_x,y_ is the number of grid cells where both species x and y are predicted to occur and N_x_ and N_y_ are the number of grid cells where respectively species x and y are predicted [36]. We apply a threshold at which habitat is considered suitable, using the average of all four species’ “Minimum training Presence logistic” threshold (=0.171).

## 3. Results

### 3.1. Model Validation

The four species’ models obtained AUC values ranged between 0.8 to 0.9. When tested against a null-model, two of our models (*M. arctoides* and *M. leonina*) performed significantly higher than random (*p* < 0.05). Comparatively, the Boyce Index validation test indicated a significant model prediction for the four species. The model for *M. mulatta* also showed a significant predicting success rate (*p* < 0.01) when evaluated with Pearson *et al.*’s jackknife method (Table 3).

**Table 3:** Model validation with test area under curve (AUC) values, null-model, Boyce Index and jackknife methods.

Species	Species-model’s test AUC	Null-model’s 95% C.I. training AUC	pValue Boyce Index (Spearman’s *rho*)	pValue Jackknife test (*D* statistic)
*M. arctoides*	0.902 *	0.783	0.000 **	–
*M. assamensis*	0.803	0.811	0.001 **	–
*M. leonina*	0.895 *	0.820	0.000 **	–
*M. mulatta*	0.779	0.817	0.039 *	0.001 **

* species-model performed better than expected by chance, *p* < 0.05. ** species-model performed better than expected by chance, *p* < 0.01.

### 3.2. Species Distribution Models Outputs

**Figure 2:**
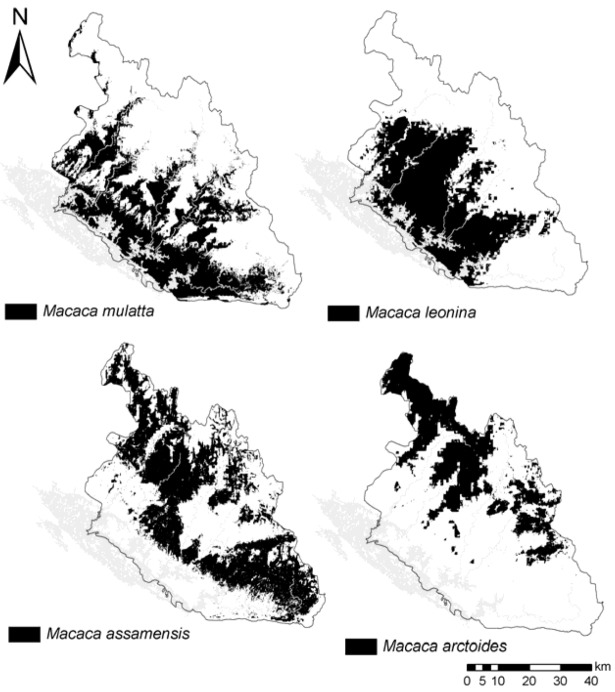
Respective predicted distribution of the four species of macaques occurring in Nakai-Nam Theun National Protected Area (NNT NPA) (predicted distribution size: *M. arctoides* 877 km^2^; *M. assamensis*: 1,476 km^2^; *M. leonina* 1,305 km^2^; *M. mulatta* 1,289 km^2^).

Predicted geographical distribution varies between the four macaque species but shows areas of overlap, in accordance with occurrence records collected (Table 4). The four species have a general gradual predicted distribution with *M. arctoides*, *M. assamensis*, *M. leonina* and *M. mulatta*, respectively along a geographic gradient (Figure 2). Both *M. assamensis* and *M. arctoides* are predicted predominantly in evergreen and mixed-deciduous forests and the farthest from village settlements (average of 13 and 16 km, respectively). *M. leonina* and *M. mulatta* are predicted mainly in evergreen and mixed broadleaf-coniferous forest. The latter two species are predicted the closest to villages (average of 6 and 8 km, respectively).

**Table 4:** Number of locations (GPS coordinate) that are common between the four species; obtained from the total occurrence points of each species used for the model (*cf.* Table 1).

**Species**	*M. arctoides*	*M. assamensis*	*M. leonina*	*M. mulatta*
*M. arctoides*	−	7	1	0
*M. assamensis*		−	0	0
*M. leonina*			−	3
*M. mulatta*				−

As per respective potential species distribution, there is a gradual change throughout the NPA in predicted mixed-species range overlap. Using the average of the four species’ minimum training presence threshold, the largest overlap is between *M. arctoides* and *M. assamensis* (overlap index: 0.77), and the smallest between *M. arctoides* with *M. mulatta* and *M. leonina* (0.12) (Table 5). The predicted distribution of *Macaca assamensis* overlaps the most (average of 0.54) with all other species, resulting from a widespread predicted distribution across the area (Table 5). 

**Table 5:** Range overlap index between the four macaque species using a threshold of presence of 0.171; range overlap between species x and y = (N_x,y_/min[N_x_, N_y_]), where N_x,y_ is the number of grid cells where both species x and y are predicted and N_x_ and N_y_ are the number of grid cells where respectively species x and y are predicted.

**Species**	*M. arctoides*	*M. assamensis*	*M. leonina*	*M. mulatta*
*M. arctoides*	−	0.77	0.12	0.12
*M. assamensis*		−	0.51	0.35
*M. leonina*			−	0.78
*M. mulatta*				−
average	**0.33**	**0.54**	**0.45**	**0.42**

**Figure 3:**
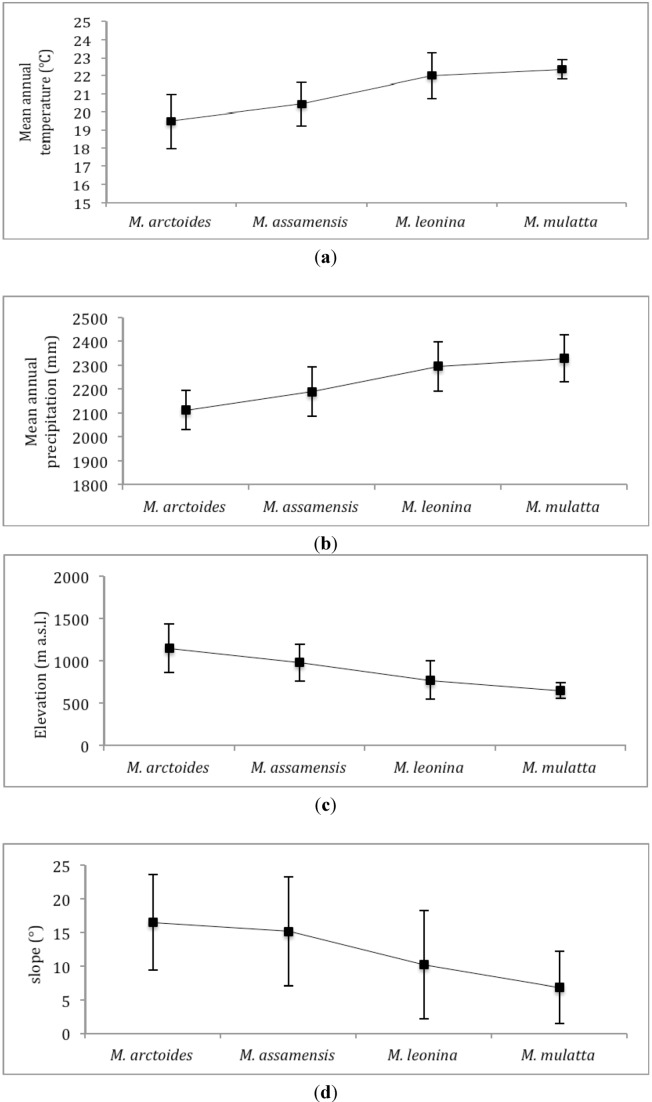
Mean (and standard deviation) annual temperature (**a**), annual precipitation (**b**), elevation (**c**) and slope (**d**) in respective predicted distribution of each macaque species.

In accordance with the predicted distribution overlaps between species, the niche similarity statistical tests indicate the niche of *M. arctoides* and *M. assamensis* as the most closely comparable, while the niche of *M. arctoides* as the most distant from the ones of *M. leonina* and *M. mulatta* (Table 6). Further to the respective gradual geographic distribution predicted, environmental characteristics within their potential distribution differ between the four species with *M. mulatta* and *M. arctoides* at the two extremes. While *M. mulatta* is modelled predominantly in a wetter and hotter niche at lower elevations and on flatter terrain, *M. arctoides* is predicted to inhabit drier and colder areas, at the highest elevations and terrain with steepest slopes (Figure 3).

**Table 6:** Pair-wise niche similarity statistical test (*D*, *I*) scores.

**Species**	**test**	*M. artoides*	*M. assamensis*	*M. leonina*	*M. mulatta*
*M. artoides*	*D*	−	0.55	0.20	0.22
	*I*	−	0.83	0.41	0.47
*M. assamensis*	*D*		−	0.39	0.40
	*I*		−	0.66	0.70
*M. leonina*	*D*			−	0.52
	*I*			−	0.79
*M. mulatta*	*D*				−
	*I*				−

## 4. Discussion

### 4.1. Macaques’ Predicted Distribution and Niches

Our potential distributions obtained from the models indicate a niche differentiation between the four species occurring in NNT NPA. The four species’ distributions alternate in parapatry and sympatry throughout the NPA as species are constrained along a gradient variation in environmental parameters. 

Potential distribution of *Macaca mulatta* is mostly predicted in lowland (mean elevation 645 m a.s.l.) and flat terrain in mixed broadleaf-coniferous and evergreen habitat, and closely associated with water sources. It is the main species for which distribution is predicted around village clusters and main rivers, which is also the case across the country [37]. *Macaca leonina* is restrained by higher elevations and colder climate further east from its predicted distribution. *Macaca assamensis* is the most widespread species across the NPA. As a result, its habitat range overlaps and shares the most similarities with all other taxa. In Laos, it is the most common macaque species in mountainous areas and is also found at lower elevations in limestone formations [9]. *Macaca arctoides* is distributed the furthest east in NNT NPA, along the Lao-Vietnam border. Its niche is characterised by the highest elevations (mean 1,098 m. a.s.l.) and the most rugged terrain. It inhabits mostly dense evergreen and mixed-deciduous forests at colder annual temperatures.

Macaques are the most widespread group of monkeys in Southeast Asia [38]. Most species from the genus have only diverged phylogenetically during the Pleistocene (~2 mya), resulting in similar morphologies observed today (brown colour, similar size; ~6.5 to 12.2 kg for our study species [23,39]). Identification success of macaques species during field surveys was relatively low, as previous researchers have found [9]. Thus, camera traps proved essential here for our modelling of their ecological niches. Despite morphological similarity, however, Fooden [17] suggested that throughout Southeast Asia macaque species are ecologically and/or geographically segregated, resulting from interspecific competition. Ever since, macaques’ ecological segregation at a local scale had rarely been studied [40,41,42] and never using a habitat suitability model.

Due to their proximity to human settlements, *M. mulatta* and *M. leonina* are at risk from hunting: macaques are hunted for food in NNT NPA and macaque bones are commonly encountered in poacher camps in the NPA [22]. As wildlife is mainly hunted using ground snares, set throughout the area [22], terrestrial *M. arctoides* is also threatened by hunting, despite being predicted as the most distant from human villages. Distance form villages is not necessarily a good predictor of hunting pressure in this case, as Vietnamese poachers commonly cross the international border for hunting [22]. Two of the four species are classified as globally threatened in the IUCN Red List (VU *M. arctoides* and VU *M. leonina*) [43]. Although species distribution models are effective to explore species’ niche, these can be subject to prediction uncertainties and should be used as baseline studies to further investigate local species’ habitat that can be validated and refined *post hoc* with additional field data [44]. Anthropogenic factors are, for instance, rarely well represented into models: species may have been extirpated from areas that have been deforested or under severe pressure of hunting. It is also important to note that the respective predicted distributions of the four species may have been influenced by the sampling bias across the area, resulting in unpredicted regions where the species may however occur [45]. Nonetheless, the use of reliable biological data for our models makes our predictions and analysis highly informative [46].

### 4.2. Model Performance

We demonstrate the value of MaxEnt in ecological studies [5,47,48] and in this case for examining the ecological nice of sympatric taxa. MaxEnt has been demonstrated as one of the best methods to handle small sample sizes [34,49]. Although the method has been improved in the past few years [28], new issues have been identified and assessed to improve the predictions of MaxEnt’s models e.g., [4,6,45,50]. We note that methods both for model selection and model validation still need to be refined. No consensus exists on variable selection methods; in any case, variables should be selected in terms of the species’ ecology and represent the complexity of the ecosystem, avoiding over- or under-parameterization [51]. We remark that the AUC value is not reliable on its own for model validation. Our four models were significantly better than random as indicated by the Boyce Index and by the jackknife method for *M. mulatta*, but only indicated a significant predictive power for two species when tested against a null-model. This highlights the uncertainties of model predictions and the need of using several validation methods in species niche modelling. The smallest AUC values for *M. assamensis and M. mulatta,* not validated by the null-model method, may be due to their ecological and geographical wider range which is commonly observed for such species [24,52].

### 4.3. Implications

In Laos and elsewhere in Southeast Asia, several species have rarely been directly observed in the field or are difficult to identify and differentiate from other taxa from signs only or when briefly sighted. Some of these species have mostly been recorded with camera traps only (e.g., saola *Pseudoryx nghetinhensis* [53]; large-antlered muntjac *Muntiacus vuquangensis* [20]; Annamite striped rabbit *Nesolagus timminsi* [54]; Sumatran striped rabbit *N. netscheri* [55]; Owston’s civet *Chrotogale owstoni* [56]; several small to medium-sized cats [57,58]). Of these species, all are classified by the IUCN [43] as globally threatened or remaining Data Deficient. Potential species distributions are crucial for species management planning of threatened species, especially in the context of biodiversity crisis well known in Southeast Asia [59,60], where “protected” status of most forested areas does not imply long-term viability and protection of species [61,62].

## 5. Conclusion

Camera-trapping can increase elusive-species records with a relatively reduced and more systematic (less biased) sampling effort [63]. These accumulated occurrence data, when combined with species distribution modelling, can be used to examine unresolved species’ niches and potential distributions, which will have crucial implications for future research and species management and conservation. Our data can be used as such by relevant management authorities and biologists. These techniques can be applied to even the most remote regions and the least known species and call for wider availability and open access.

## References

[1] Bojórquez-Tapia L.A., Azuara I., Ezcurra E., Flores-Villela O. (1995). Identifying conservation priorities in Mexico through geographic information systems and modeling. Ecol. Appl..

[2] Raxworthy C.J., Ingram C.M., Rabibisoa N., Pearson R.G. (2007). Applications of ecological niche modeling for species delimitation: A review and empirical evaluation using day geckos (*Phelsuma*) from Madagascar. Syst. Biol..

[3] Buermann W., Saatchi S., Smith T.B., Zutta B.R., Chaves J.A., Milá B., Graham C.H. (2008). Predicting species distributions across the Amazonian and Andean regions using remote sensing data. J. Biogeogr..

[4] Parolo G., Rossi G., Ferrarini A. (2008). Toward improved species niche modelling: Arnica montana in the Alps as a case study. J. Appl. Ecol..

[5] Martínez-Freiría F., Sillero N., Lizana M., Brito J.C. (2008). GIS-based niche models identify environmental correlates sustaining a contact zone between three species of European vipers. Divers. Distrib..

[6] Pineda E., Lobo J.M. (2009). Assessing the accuracy of species distribution models to predict amphibian species richness patterns. J. Appl. Ecol..

[7] Timmins R.J., Evans T.D., Khounboline K., Sisomphone C. (1998). Status and conservation of the giant muntjac *Megamuntiacus vuquangensis*, and notes on other muntjac species in Laos. Oryx.

[8] Duckworth J.W., Stones T., Tizard R., Watson S., Wolstencroft J. (2010). Does the fishing cat inhabit Laos?. Cat News.

[9] Timmins R.J., Duckworth J.W. (2011). Distribution and habitat of Assamese Macaque *Macaca assamensis* in Lao PDR, including its use of low altitude karsts. Primate Conserv..

[10] Duckworth J.W., Poole C.M., Tizard R.J., Walston J.L., Timmins R.J. (2005). The jungle cat *Felis chaus* in Indochina: A threatened population of a widespread and adaptable species. Biodivers. Conserv..

[11] Tobler M.W., Carrillo-Percastegui S.E., Leite Pitman R., Mares R., Powell G. (2008). An evaluation of camera traps for inventorying large- and medium-sized terrestrial rainforest mammals. Anim. Conserv..

[12] Moruzzi T.L., Fuller T.K., DeGraaf R.M., Brooks R.T., Li W. (2002). Assessing remotely triggered cameras for surveying carnivore distribution. Wildl. Soc. Bull..

[13] Di Bitetti M.S., De Angelo C.D., Di Blanco Y.E., Paviolo A. (2010). Niche partitioning and species coexistence in a Neotropical felid assemblage. Acta Oecol..

[14] Pereira P., da Silva A.A., Alves J., Matos M., Fonseca C. (2012). Coexistence of carnivores in a heterogeneous landscape: Habitat selection and ecological niches. Ecol. Res..

[15] Jenks K.E., Kitamura S., Lynam A.J., Ngoprasert D., Chutipong W., Steinmetz R., Sukmasuang R., Grassman L.I., Cutter P., Tantipisanuh N., Bhumpakphan N., Gale G.A., Reed D.H., Leimgruber P., Songsasen N. (2012). Mapping the distribution of dholes, *Cuon alpinus* (Canidae, Carnivora), in Thailand. Mammalia.

[16] Timmins R.J., Evans T.D. (1996). Wildlife and Habitat Survey of the Nakai-Nam Theun National Biodiversity Conservation Area.

[17] Fooden J. (1982). Ecogeographic segregation of macaque species. Primates.

[18] Robichaud W., Sinclair A., Odarkor-Lanquaye N., Klinkenberg B. (2009). Stable forest cover under increasing populations of swidden cultivators in central Laos: The roles of intrinsic culture and extrinsic wildlife trade. Ecol. Soc..

[19] Hijmans R.J., Cameron S.E., Parra J.L., Jones P.G., Jarvis A. (2005). Very high resolution interpolated climate surfaces for global land areas. Int. J. Climatol..

[20] Johnson A., Johnston J. (2007). Biodiversity Monitoring and enforcement project in the Nam Theun 2 watershed (including the Nakai-Nam Theun NPA and corridors linking to the Phou Hin Poun and Hin Nam Nor PA).

[21] NT2-WMPA (Nam Theun 2-Watershed Management Protection Authority) (2011). Camera-Trap Database 2006–2011.

[22] Coudrat C.N.Z. (2012). Wildlife Surveys in Nakai-Nam Theun National Protected Area 2011-2012 (Final Report).

[23] Thierry B., Campbell C., Fuentes A., MacKinnon K., Bearder S., Stumpf R. (2010). The macaques. Primates in Perspective.

[24] Phillips S.J., Dudík M., Schapire R.E. A maximum entropy approach to species distribution modeling. Proceedings of the Twenty-First International Conference on Machine Learning.

[25] Phillips S.J., Anderson R.P., Schapire R.E. (2006). Maximum entropy modeling of species geographic distributions. Ecol. Model..

[26] Elith J., Ferrier S., Guisan A., Graham C.H., Anderson R.P., Dudı M., Hijmans R.J., Huettmann F., Leathwick J.R., Lehmann A., Li J., Lohmann L. G., Loiselle B.A., Manion G., Moritz C., Nakamura M., Nakazawa Y., Overton J.M., Peterson A.T., Phillips S.J., Richardson K., Scachetti-pereira R., Schapire R.E., Williams S., Wisz M.S., Zimmermann N.E. (2006). Novel methods improve prediction of species distributions from occurrence data. Ecography.

[27] Kohavi R. A study of cross-validation and bootstrap for accuracy estimation and model selection. Proceedings of the Fourteenth International Joint Conference on Artificial Intelligence.

[28] Phillips S.J., Dudik M. (2008). Modeling of species distributions with Maxent: New extensions and a comprehensive evaluation. Ecogeography.

[29] Raes N., Steege H. (2007). A null-model for significance testing of presence-only species distribution models. Ecography.

[30] Boyce M.S., Vernier P.R., Nielsen S.E., Schmiegelow F.K. (2002). Evaluating resource selection functions. Ecol. Model..

[31] Pearson R.G., Raxworthy C.J., Nakamura M., Townsend Peterson A. (2007). Predicting species distributions from small numbers of occurrence records: A test case using cryptic geckos in Madagascar. J. Biogeogr..

[32] Yost A.C., Miller R. (2008). Predictive modeling and mapping sage grouse (*Centrocercus urophasianus*) nesting habitat using Maximum Entropy and a long-term dataset from Southern Oregon. Ecol. Inform..

[33] Hirzel A.H., Le Lay G., Helfer V., Randin C., Guisan A. (2006). Evaluating the ability of habitat suitability models to predict species presences. Ecol. Model..

[34] Hernandez P.A., Graham C.H., Master L.L., Albert D.L. (2006). The effect of sample size and species characteristics on performance of different species distribution modeling methods. Ecogeography.

[35] Schoener T.W. (1968). Anolis lizards of Bimini: Resource partitioning in a complex fauna. Ecology.

[36] Warren D.L., Glor R.E., Turelli M. (2008). Environmental niche equivalency versus conservatism: Quantitative approaches to niche evolution. Evolution.

[37] Duckworth J., Salter R.E., Khounboline K. (1999). Wildlife in Lao PDR 1999 Status Report.

[38] Abegg C., Thierry B. (2002). Macaque evolution and dispersal in insular south-east Asia. Biol. J. Linn. Soc..

[39] Eudey A.A., Lindburg D.G. (1980). Pleistocene glacial phenomena and the evolution of Asian macaques. The Macaques: Studies in Ecology, Behavior and Evolution.

[40] Eudey A.A., Ehara A., Kimura T., Takenaka O., Iwamoto M. (1991). Macaques habitat preference in west-central Thailand and quaternary glacial events. Primatology Today.

[41] Rodman P.S. (1991). Structural Differentiation of microhabitats of sympatric *Macaca fascicularis* and *M. nemestrina* in East Kalimantan, Indonesia. Int. J. Primatol..

[42] Borries C., Larney K., Kreetiyutanony K., Koenig A. (2002). The diurnal primate community in a dry evergreen forest in Phu Khieo Wildlife Sanctuary, Northeast Thailand. Nat. Hist. Bull. Siam Soc..

[43] IUCN Red List v.2011. http://www.iucnredlist.org.

[44] Wintle B.A., Elith J., Potts J.M. (2005). Fauna habitat modelling and mapping: A review and case study in the Lower Hunter Central Coast region of NSW. Austral. Ecol..

[45] Phillips S.J., Dudík M., Elith J., Graham C.H., Lehmann A., Leathwick J., Ferrier S. (2009). Sample selection bias and presence-only distribution models: Implications for background and pseudo-absence data. Ecol. Appl..

[46] Blanco G., Sergio F., Sanchéz-Zapata J.A., Pérez-García J.M., Botella F., Martínez F., Zuberogoitia I., Frías O., Roviralta F., Martínez J.E., Hiraldo F. (2012). Safety in numbers? Supplanting data quality with fanciful models in wildlife monitoring and conservation. Biodivers. Conserv..

[47] Thorn J.S., Nijman V., Smith D., Nekaris K.A.I. (2009). Ecological niche modelling as a technique for assessing threats and setting conservation priorities for Asian slow lorises (Primates: Nycticebus). Divers. Distrib..

[48] Chunco A.J., Jobe T., Pfennig K.S. (2012). Why do species co-occur? A test of alternative hypotheses describing abiotic differences in sympatry versus allopatry using spadefoot toads. PLoS ONE.

[49] Wisz M.S., Hijmans R., Li J., Peterson A.T., Graham C.H., Guisan A., The Nceas Predicting Species Distributions Working Group (2008). Effects of sample size on the performance of species distribution models. Divers. Distrib..

[50] Graham C.H., Elith J., Hijmans R.J., Guisan A., Townsend-Peterson A., Loiselle B.A., The Nceas Predicting Species Distributions Working Group (2008). The influence of spatial errors in species occurrence data used in distribution models. J. Appl. Ecol..

[51] Warren D., Steifert S. (2011). Ecological niche modeling in Maxent: The importance of model complexity and the performance of model selection criteria. Ecol. Appl..

[52] McPherson J.M., Jetz W. (2007). Effects of species? Ecology on the accuracy of distribution models. Ecography.

[53] Hardcastle J., Cox S., Dao N.T., Johns A.G. Rediscovering the Saola. Proceedings of the Rediscovering the Saola—A Status Review and Conservation Planning Workshop.

[54] Surridge A.K., Timmins R.J., Hewitt G.M., Bell D.J. (1999). Striped rabbits in Southeast Asia. Nature.

[55] McCarthy J.L., Fuller T.K., McCarthy K.P., Wibisono H.T., Livolsi M.C. (2012). Using camera trap photos and direct sightings to identify possible refugia for the Vulnerable Sumatran striped rabbit *Nesolagus netscheri*. Oryx.

[56] Johnson A., Vongkhamheng C., Hedemark M., Saithongdam T. (2006). Effects of human carnivore conflict on tiger (*Panthera tigris*) and prey populations in Lao PDR. Anim. Conserv..

[57] Mohamed A., Samejima H., Wilting A. (2009). Records of five Bornean cat species from Deramakot Forest Reserve in Sabah, Malaysia. Cat News.

[58] Wilting A., Mohamed A., Ambu L.N., Lagan P., Mannan S., Hofer H., Sollmann R. (2012). Density of the Vulnerable Sunda clouded leopard *Neofelis diardi* in two commercial forest reserves in Sabah, Malaysian Borneo. Oryx.

[59] Sodhi N.S., Koh L.P., Brook B.W., Ng P.K.L. (2004). Southeast Asian biodiversity: An impending disaster. Trends Ecol. Evol..

[60] Duckworth J.W., Batters G., Belant J.L., Bennett E.L., Brunner J., Burton J., Challender D.W.S., Cowling V., Duplaix N., Harris J.D., Hedges S., Long B., Mahood S.P., McGowan P.J.K., McShea W.J., Oliver W.L.R., Perkin S., Rawson B.M., Shepherd C.R., Stuart S.N., Talukdar B.K., van Dijk P.P., Vié J.-C., Walston J.L., Whitten T., Wirth R. (2012). Why South-East Asia should be the world’s priority for averting imminent species extinctions, and a call to join a developing cross-institutional programme to tackle this urgent issue. SAPIENS.

[61] Catullo G., Masi M., Falcucci A., Maiorano L., Rondinini C., Boitani L. (2008). A gap analysis of Southeast Asian mammals based on habitat suitability models. Biodivers. Conserv..

[62] Yen P., Ziegler S., Huettmann F., Onyeahialam A.I. (2005). Change detection of forest and habitat resources from 1973 to 2001 in Bach Ma National Park, Vietnam, using remote sensing imagery. Int. For. Rev..

[63] Kelly M.J. (2008). Design, evaluate, refine: Camera trap studies for elusive species. Anim. Conserv..

